# Value of ^18^ F-FDG PET/CT to Identify Occult Infection in Presumed Aseptic Pseudarthrosis after Spinal Fusion: Correlation with Intraoperative Cultures

**DOI:** 10.1055/s-0044-1778711

**Published:** 2024-01-22

**Authors:** Yacine El Yaagoubi, Eric Lioret, Clément Thomas, Jean-Edouard Loret, Adrien Simonneau, Anne-Victoire Michaud-Robert, Laurent Philippe, Maja Ogielska, Caroline Prunier-Aesch

**Affiliations:** 1 Department of Nuclear Medicine, Vinci Clinic, Tours, France; 2 Department of Neurosurgery, Vinci Clinic, Tours, France; 3 Department of Neurosurgery, NCT+ Clinic, Tours, France; 4 Department of Infectious Diseases, Vinci Clinic, Tours, France

**Keywords:** fluorine-18 fluorodeoxyglucose, positron emission tomography/computed tomography, spinal fusion, surgical site infection, pseudarthrosis, *Cutibacterium acnes*, coagulase-negative *staphylococci*

## Abstract

**Objective** Fluorine-18 fluorodeoxyglucose ( ^18^ F-FDG) positron emission tomography/computed tomography (PET/CT) has gained attention as an emerging tool in case of suspicion of infection on spine, whether native or instrumented. However, the diagnostic performance of ^18^ F-FDG PET/CT in clinically occult low-grade surgical site infection (SSI) after spinal fusion, an important risk factor for pseudarthrosis, remains unknown.

**Methods** We retrospectively identified all the presumed aseptic patients with pseudarthrosis confirmed by revision surgery who underwent preoperative ^18^ F-FDG PET/CT scans performed between April 2019 and November 2022. These patients were presumed aseptic because they did not have clinical signs or laboratory tests suggestive of SSI, preoperatively. The PET/CT images were analyzed in consensus by two nuclear medicine physicians blinded to the clinical, biological, and imaging information. Visual assessment of increased uptake around cage/intervertebral disk space (and/or hardware) higher than background recorded from the first normal adjacent vertebra was interpreted as positive. Image data were also quantitatively analyzed by the maximum standardized uptake value as an index of ^18^ F-FDG uptake, and the ratio between the uptake around cage/intervertebral disk space (and/or hardware) and background recorded from the first normal adjacent vertebra was calculated. The final diagnosis of infection was based on intraoperative cultures obtained during pseudarthrosis revision surgery.

**Results** Thirty-six presumed aseptic patients with surgically confirmed pseudarthrosis after spinal fusion underwent preoperative ^18^ F-FDG PET/CT scans. Cultures of samples from revisions found that 20 patients (56%) were infected. The most frequent isolated bacterium was *Cutibacterium acnes* ( *C. acnes* ) in 15 patients (75%), followed by coagulase-negative *staphylococci* (CNS) in 7 patients (33%). Two patients had co-infections involving both *C. acnes* and CNS. Of the 36 PET/CT studied in this study, 12 scans were true-negative, 10 true-positive, 10 false-negative, and 4 false-positive. This resulted in sensitivity, specificity, positive predictive value, negative predictive value, and diagnostic accuracy of 50%, 75%, 71%, 55%, and 61%, respectively.

**Conclusion** In presumed aseptic pseudarthrosis after spinal fusion, ^18^ F-FDG PET/CT offers good specificity (75%) but low sensitivity (50%) to identify occult SSI. The high prevalence (56%) of SSI, mostly caused by *C. acnes* (75%), found in our presumed aseptic cohort of patients supports the utility of systematic intraoperative cultures in revision cases for pseudarthrosis.

## Introduction

Pseudarthrosis is a well-known cause of persistent or recurrent pain after spinal fusion surgery, and may occur in up to 40% of cases. Symptomatic pseudarthrosis is defined as the absence of solid bony fusion, at a minimum follow-up of 6 months after spinal surgery, and resultant mechanical back pain. Revision surgery is the preferred treatment in patients suffering from symptomatic pseudarthrosis. 1 Along with spinal imbalance, insufficient primary implant construct stability, osteoporosis, smoking, and long-term steroid use, low-grade infection has been identified as an important risk factor for pseudarthrosis. 2 3 This relatively new clinical entity in the spine has been widely discussed in the shoulder literature. 4

Pseudarthrosis caused by low-virulence bacteria is often presumed to be aseptic because of its delayed presentation and often normal inflammatory markers (blood counts, erythrocyte sedimentation rate [ESR], C-reactive protein [CRP]). 5 Because surgical site infection (SSI) requires a different surgical strategy (i.e., thorough debridement) and immediate start of antibiotic preoperatively, it is crucial to identify SSI prior to revision surgery. 6 Magnetic resonance imaging (MRI) is considered the gold standard imaging method when spinal infection is suspected. However, MRI diagnostic accuracy is limited in the postoperative spine by the nonspecific signal characteristics, reflecting either active infection or reparative tissue processes. 7 Moreover, metallic artifacts from implant material can also negatively affect its diagnostic accuracy. 8

In the literature, several studies have reported the usefulness of fluorine-18 fluorodeoxyglucose ( ^18^ F-FDG) positron emission tomography/computed tomography (PET/CT) in case of suspicion of spinal infection, suggesting the possible dominance of ^18^ F-FDG PET/CT over MRI. Some authors even reported a negative predictive value (NPV) close to 100% of ^18^ F-FDG PET/CT in spinal infection and concluded that a negative ^18^ F-FDG PET/CT can potentially exclude infection. 9 10 11 Recently, the second International Consensus Meeting on Musculoskeletal Infection recommended the use of ^18^ F-FDG PET/CT as an adjunct to MRI to diagnose spinal infections when an MRI cannot be performed or is inconclusive. 12 Regarding the postoperative spine, few studies suggested the usefulness of ^18^ F-FDG PET/CT in patients with suspected SSI, having an elevated blood count or CRP level, with or without the presence of high fever. 13 14 15

However, the diagnostic performance of ^18^ F-FDG PET/CT in clinically occult SSI after spinal fusion remains unknown. The aim of our study was to determine the diagnostic accuracy of ^18^ F-FDG PET/CT to identify occult infection in presumed aseptic patients with confirmed pseudarthrosis on revision surgery. Validation of ^18^ F-FDG PET/CT results was based on microbiological findings obtained from intraoperative cultures.

## Materials and Methods

### Patients

This compliant study received a local institutional review board approval. Written informed consent was waived due to the retrospective nature of this study, and the data were anonymized. We identified all the presumed aseptic patients with pseudarthrosis confirmed by revision surgery who underwent preoperative ^18^ F-FDG PET/CT scans performed between April 2019 and November 2022. These patients were presumed aseptic because they did not have clinical signs or laboratory tests suggestive of SSI, preoperatively. All the ^18^ F-FDG PET/CT scans were performed more than 12 months after initial spinal fusion surgery. A total of 36 patients (23 women, 13 men; age range: 30–73 years) met the inclusion criteria. Location of the initial spinal fusion, the number of fused levels, the type of surgery, and material were recorded. Regarding biological characteristics, blood counts (white blood cell, blood polymorphonuclear neutrophil) were obtained from routine preoperative tests ( Table 1 ). CRP was not available since there was no suspicion of SSI preoperatively.

**Table 1:** Patient data

	Global population ( *n* = 36)	Infected ( *n* = 20)	Noninfected ( *n* = 16)	*p* -Value
Location, *n* (%)				
Cervical	18 (50)	11 (55)	7 (44)	
Lumbar	12 (33)	7 (35)	5 (31)	
Thoraco-lumbar	1 (3)	1 (5)	0 (0)	
Lumbo-sacral	5 (14)	1 (5)	4 (25)	
Number of fused levels, *n* (%)				
≤ 1	25 (69)	10 (50)	15 (94)	
≥ 2	11 (31)	10 (50)	1 (6)	
Type of surgery, *n* (%)				
Intersomatic cage(s)	24 (67)	14 (70)	10 (62)	
Arthrodesis with intersomatic cage(s)	9 (25)	3 (15)	6 (38)	
Arthrodesis without intersomatic cage(s)	3 (8)	3 (15)	0 (0)	
Type of material, *n* (%)				
PEEK	25 (69)	14 (70)	11 (69)	
PEEK + metal	8 (22)	3 (15)	5 (31)	
Metal	3 (8)	3 (15)	0 (0)	
Time between PET/CT and revision surgery (months), mean (median)	4 (2.8)	3.1 (2.5)	5.2 (3.4)	0.2
Biological characteristics				
White blood cell, mean (SD) G/L	7.8 (2.6)	8.2 (3.2)	7.6 (1.8)	0.63
Blood polymorphonuclear neutrophil, mean (SD) G/L	4.8 (1.8)	4.9 (2.3)	4.7 (1.4)	0.84
PET/CT characteristics				
Positive visual assessment, *n* (%)	14 (39)	10 (50)	4 (25)	
SUVmax, mean (SD)	3.7 (2.2)	4.1 (0.7)	3.3 (1.8)	0.31
Uptake ratio, mean (SD)	1.3 (0.7)	1.4 (0.7)	1.1 (0.5)	0.16

Abbreviations: PEEK, polyetheretherketone; PET/CT, positron emission tomography/computed tomography; SD, standard deviation; SUVmax, maximum standardized uptake value.

### Gold Standard

During pseudarthrosis revision surgery, at least three different samples from bone tissue and/or extracted hardware were taken for microbiological investigations. Microbiological samples were incubated for 14 days for aerobic and anaerobic cultures. To rule out the possibility of contamination, diagnosis of occult SSI was made if at least two positive intraoperative cultures of the same pathogen were isolated. 16

### Scanning

All patients underwent PET/CT imaging after fasting for at least 6 hours and with capillary glycemia lower than 11 mmol/L. The acquisition was made 60 minutes after ^18^ F-FDG intravenous injection (3 MBq/kg). The PET/CT images were obtained using an integrated PET/CT scanner (Discovery IQ; GE-Healthcare, Milwaukee, Wisconsin, United States). After a low-dose CT acquisition (120 kV, 30 mAs, slice thickness 4 mm) for attenuation correction, three-dimensional PET scan from midthigh to vertex was acquired at 2 minute/bed position. This was immediately followed by a noncontrast-enhanced diagnostic CT scan (16-slice helical, 100–140 kV, 80–200 mAs, 2.5 mm slice thickness). The fused ^18^ F-FDG PET/CT images were displayed in axial, sagittal, and coronal slices.

### Interpretation

The PET/CT images were visually reviewed using Advantage Window Volume Viewer software (GE-Healthcare, Milwaukee, Wisconsin, United States), providing multiplanar reformatted images of PET alone, CT alone, and fused PET/CT. Images were analyzed in consensus by two board-certified nuclear medicine physicians (YEY and CPA), who were blinded to the clinical, biological, and imaging information. Attenuation-corrected PET images as well as fused PET/CT images were used for analysis, using the CT for anatomical correlation. Visual assessment of increased uptake around cage/intervertebral disk space (and/or hardware) higher than background recorded from the first normal adjacent vertebra was interpreted as positive. Image data were also quantitatively analyzed by the maximum standardized uptake value (SUVmax) as an index of ^18^ F-FDG uptake, and the ratio between the uptake around cage/intervertebral disk space (and/or hardware) and background recorded from the first normal adjacent vertebra was calculated.

### Statistical Analysis

IBM SPSS statistics version 29 (SPSS Inc., Chicago, Illinois, United States) was used for statistical analysis. *p* -Values less than 0.05 were considered statistically significant. Differences between groups were calculated using the Mann–Whitney U test. The sensitivity, specificity, positive predictive value (PPV), NPV, and diagnostic accuracy were calculated using visual assessment versus true infection as indicated in the definition for SSI in the text above.

## Results

### Patient Characteristics

A total of 36 presumed aseptic patients with surgically confirmed pseudarthrosis after spinal fusion underwent preoperative ^18^ F-FDG PET/CT scans (23 women, 13 men; age range: 30–73 years) during the study period ( Table 1 ). Cultures of samples from revisions found that 20 patients (56%) of these presumed aseptic patients were infected. The most frequent isolated bacterium was *Cutibacterium* *acnes* in 15 patients (75%), followed by coagulase-negative *staphylococci* (CNS) in 7 patients (33%), *Staphylococcus epidermidis* in 3 patients (14%), *Staphylococcus saccharolyticus* in 2 patients (9.5%), *Staphylococcus capitis* in 2 patients (9.5%), *and Staphylococcus hominis* in 1 patient (4.8%). It is worth noting that intraoperative cultures grew *C. acnes* , *S. epidermidis* , and *S. capitis* in one patient and *C. acnes* associated with *S. saccharolyticus* in another patient ( Table 2 ). Concerning biological characteristics, there was no statistically significant difference in white blood cell and blood polymorphonuclear neutrophil values between the infected and noninfected groups ( *p* = 0.63 and *p* = 0.84, respectively; Table 1 ).

**Table 2:** Microbiological findings obtained from intraoperative cultures in the infected group

	Occult infections ( *n* = 20) a
Microorganisms	
*Cutibacterium acnes*	15 (75%)
Coagulase-negative staphylococci	
*Staphylococcus epidermidis*	3 (14%)
*Staphylococcus saccharolyticus*	2 (9.5%)
*Staphylococcus capitis*	2 (9.5%)
*Staphylococcus hominis*	1 (4.8%)

a Intraoperative cultures grew *Cutibacterium acnes* and *Staphylococcus epidermidis* and *Staphylococcus capitis* in one patient; and *Cutibacterium acnes* and *Staphylococcus saccharolyticus* in one patient.

### PET/CT Results

**Fig. 1:**
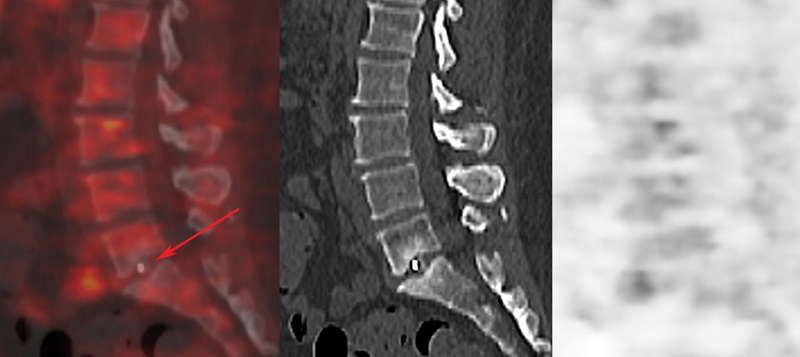
Fluorine-18 fluorodeoxyglucose ( ^18^ F-FDG) positron emission tomography/computed tomography (PET/CT) fusion, noncontrast CT, and PET images in a true-negative patient 16 months after spinal fusion. Sagittal PET/CT showed no uptake of the tracer around cage at L5-S1 ( *red arrow* ). The four intraoperative cultures obtained during pseudarthrosis revision were negatives.

Of the 36 PET/CT studied in this study, 12 scans were true-negative, 10 true-positive, 10 false-negative, and 4 false-positive ( Figs. 1 2 3 4 ). This resulted in sensitivity, specificity, PPV, NPV, and diagnostic accuracy of 50%, 75%, 71%, 55%, and 61%, respectively. Results are summarized in Tables 3 and 4 . SUVmax and uptake ratio values were not statistically different between the infected and noninfected groups ( *p* = 0.31 and *p* = 0.16, respectively; Table 1 ).

**Table 3:** FDG-PET diagnosis using visual assessment versus true infection

	FDG-PET diagnosis	
Infection based on intraoperative cultures	Negative	Positive
No infection	12	4
Infection	10	10

Abbreviation: FDG, fluorodeoxyglucose; PET, positron emission tomography.

**Table 4:** Diagnostic value of FDG-PET using visual assessment

	PET/CT visual assessment
Sensitivity (%)	50
Specificity (%)	75
Positive predictive value (%)	71
Negative predictive value (%)	55
Accuracy (%)	61

Abbreviations: CT, computed tomography; FDG, fluorodeoxyglucose; PET, positron emission tomography.

**Fig. 2:**
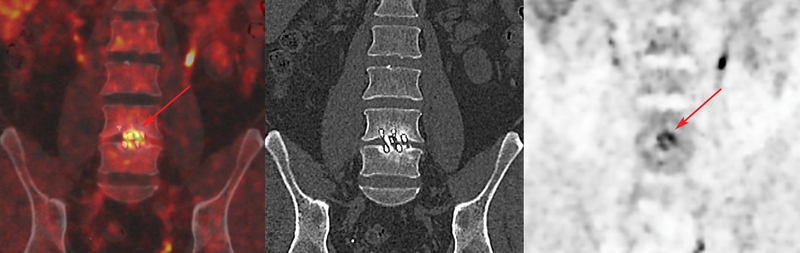
Fluorine-18 fluorodeoxyglucose ( ^18^ F-FDG) positron emission tomography/computed tomography (PET/CT) fusion, noncontrast CT, and PET images in a true-positive patient 17 months after spinal fusion. Coronal PET/CT showed increased uptake around cage at L4-L5 ( *red arrow* , SUVmax = 6.3, uptake ratio = 2). Four of four intraoperative cultures taken during pseudarthrosis revision grew *Cutibacterium acnes* .

**Fig. 3:**
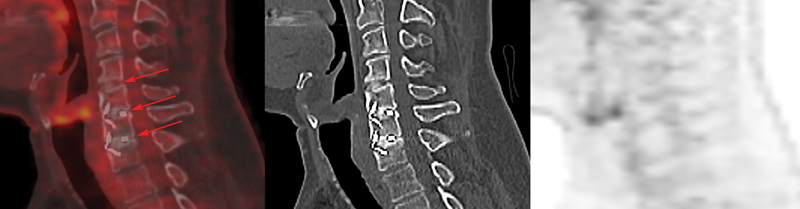
Fluorine-18 fluorodeoxyglucose ( ^18^ F-FDG) positron emission tomography/computed tomography (PET/CT) fusion, noncontrast CT, and PET images in a false-negative patient 38 months after spinal fusion. Sagittal PET/CT showed no uptake of the tracer around cages at C4-C5, C5-C6, and C6-C7 ( *red arrows* ). Five of five intraoperative cultures obtained during pseudarthrosis revision grew *Cutibacterium acnes* .

**Fig. 4:**
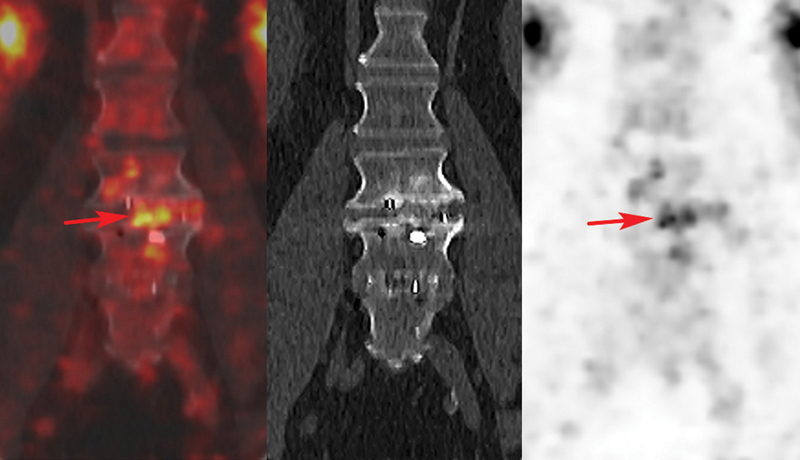
Fluorine-18 fluorodeoxyglucose ( ^18^ F-FDG) positron emission tomography/computed tomography (PET/CT) fusion, noncontrast CT, and PET images in a false-positive patient 16 months after spinal fusion. Coronal PET/CT showed increased uptake around cage at L3-L4 ( *red arrow* , SUVmax = 7.3, uptake ratio = 2.1). The three intraoperative cultures obtained during pseudarthrosis revision were negatives.

## Discussion

This study aimed to investigate the usefulness of ^18^ F-FDG PET/CT to identify occult SSI after spinal fusion in presumed aseptic patients with surgically confirmed pseudarthrosis. The main finding of our study is that ^18^ F-FDG PET/CT, in spite of a good specificity (75%), yields a low sensitivity (50%) to detect occult SSI in presumed aseptic patients, mostly caused by low-virulence bacteria *C. acnes* (75%).

SSI is a well-known risk factor for pseudarthrosis after spinal fusion. 3 SSI after spinal fusion can clinically be “occult” and its diagnosis can be challenging, especially when it is caused by low-virulence bacteria such as *C. acnes* and CNS. 5 Because surgical treatment of SSI can be complex and at risk of inducing complications, it is critical to carry out precise diagnosis prior to revision surgery. 6 14 However, no diagnostic algorithm exists to diagnose or exclude SSI after spinal fusion before revision surgery. In prosthetic joints, it is usually possible to aspirate synovial fluid to exclude an infection using cytology, biomarkers, and/or culture. 17 In spines, there is no fluid to aspirate. Thus, specific diagnostic tests remain necessary in this clinical setting.

SSI after spinal fusion surgery caused by low-virulence bacteria is difficult to detect because patients may have an indolent clinical picture. Back pain is often the main symptom reported, and most of patients are afebrile. Moreover, level of ESR and CRP may be normal or only slightly elevated, and the absence of inflammatory markers cannot rule out infection. 18 Thus, complementary medical imaging can be necessary. MRI remains the gold standard imaging technique when spinal infection is suspected because it can show pathological abnormalities in the disk and adjacent bone marrow, as well as in soft tissue. 19 However, MRI diagnostic accuracy is limited in the postoperative spine by the nonspecific signal characteristics, reflecting either active infection or reparative tissue processes, and by metallic artifacts from implant material. 7 8

^18^ F-FDG PET/CT was initially developed as a novel molecular imaging method in clinical oncology. More recently, this technique emerged as an interesting tool in several infectious and inflammatory diseases. 20 Several studies have indicated the usefulness of ^18^ F-FDG PET/CT in spinal infection, suggesting that PET/CT may be superior to MRI. Some authors even reported an NPV close to 100% and concluded that a negative ^18^ F-FDG PET/CT scan can potentially exclude infection. 9 10 11 Recently, the second International Consensus Meeting on Musculoskeletal Infection recommended the use of ^18^ F-FDG PET/CT as an adjunct to MRI to diagnose spinal infections when an MRI cannot be performed or is inconclusive. 12 Regarding the postoperative spine, few studies suggested the usefulness of ^18^ F-FDG PET/CT when SSI is suspected by clinical signs and/or laboratory parameters (elevated blood count and/or CRP level). 13 14 15 Inanami et al reported in a case control study that ^18^ F-FDG PET/CT was effective to identify infection in all eight infected patients despite the presence of spinal instruments, whereas artifacts of spinal implants rendered the MRI images unclear. 13 Follenfant et al, in their retrospective study of 44 patients, indicated that ^18^ F-FDG PET/CT was useful for the diagnosis of SSI with a sensitivity, specificity, PPV, and NPV of 86.4%, 81.5%, 79.2%, and 88.0%, respectively. 14 In a recent retrospective study of 52 subjects who underwent spine surgery, Segawa et al even found excellent diagnostic yields with sensitivity, specificity, and accuracy close to 100%. 15

We reported in our study four false-positives with ^18^ F-FDG PET/CT. Two of them concerned intersomatic polyetheretherketone (PEEK) cages and two were arthrodesis with intersomatic PEEK cages. Because all of our patients underwent spinal fusion more than 12 months before the scan, we could not assume that postoperative changes could be the cause of a local inflammation detected by ^18^ F-FDG PET/CT. As suggested by De Winter et al, we believe that possible instability of material in this context of pseudarthrosis may have created an inflammatory reaction mimicking infection on ^18^ F-FDG PET/CT. 21 On the other hand, among the 20 infected patients included in our study, 10 patients had false-negative ^18^ F-FDG PET/CT. This high false-negative rate explains the low sensitivity of ^18^ F-FDG PET/CT that we observed in our study, in spite of a good specificity. Among the 10 false-negative patients, the causative pathogen was *C. acnes* in 6 patients, *S. epidermidis* in 1 patient, *S. capitis* in 1 patient, *S. hominis* in 1 patient, and *C. acnes* associated with *S. epidermidis* and *S. capitis* in 1 patient. Such modest results suggest that alterations of ^18^ F-FDG uptake in SSI caused by low-grade infections are discrete, as demonstrated also for chronic shoulder periprosthetic joint infection. 22 In our false-negative patients, absence of ^18^ F-FDG uptake, which reflect glucose metabolism, may be explained by the low virulence of bacteria like *C. acnes* and CNS. In a study of foreign-body-associated infection in a rabbit model, Lankinen et al found that ^18^ F-FDG uptake was lower in infection with the low-virulence bacteria *S. epidermidis* compared with the highly virulent *Staphylococcus aureus* . 23 Interestingly, Follenfant et al reported that two of their three false-negative cases concerned infection with slow-growing organisms ( *C. acnes* and *S. epidermidis* ). 14 A case report by Bolander et al also described a case of a false-negative ^18^ F-FDG PET/CT in a patient with an implant-associated infection with *C. acnes* . 24

Consequently, a negative PET/CT cannot rule out confidently low-grade SSI prior to pseudarthrosis revision in presumed aseptic patients. We believe that ^18^ F-FDG PET/CT should be reserved to cases when SSI is suspected by clinical examination and/or laboratory tests. Future studies should focus on finding more sensitive diagnostic modalities such as a novel specific radiotracer to identify low-grade SSI. In the recent nuclear medicine literature, ^68^ Ga-citrate PET/CT has gained attention as a promising radiotracer to identify bone infections. In a study including 39 patients with hip and knee prosthesis, Xu et al reported that ^68^ Ga-citrate PET/CT may be able to differentiate periprosthetic joint infection from aseptic loosening. 25 However, these encouraging results should be evaluated in a larger prospective series including patients with low-grade SSI after spinal fusion.

In our presumed aseptic cohort of patients with pseudarthrosis, we found a high prevalence (56%) of unexpected SSI caused by low-virulence bacteria. The causative pathogens isolated in these SSI were *C. acnes* (75%) and CNS (33%). These data reflect the most common pathogens detected after spinal hardware removal for low-grade infection indicated in the literature. 26 27 Moreover, we reported co-infection involving *C. acnes* associated with CNS in two patients, which is also consistent with the findings of previous studies. 28 Interestingly, a recent study suggested that under anaerobic condition a *C. acnes* biofilm may participate to staphylococci colonization by providing an ideal growth environment. 29 Occult chronic low-grade periprosthetic joint infection caused by *C. acnes* has been widely discussed in the shoulder literature 4 although it is not clear whether positive cultures constantly translate into clinical infection. 30 More recently, *C. acnes* , because of its low virulence, has been reported as an increasingly prevalent pathogen in presumed aseptic pseudarthrosis, suggesting that ongoing infection may affect local osteogenesis. 16 26 In a retrospective review of 578 revision surgeries, Shifflett et al reported that *C. acnes* grew in 54.2% of cases with the primary diagnosis of aseptic pseudarthrosis, suggesting that, in revision surgery, cultures should be held for *C. acnes* in all revision cases for pseudarthrosis. 16 Burkhard et al indicated that 10.2% of revision cases for presumed aseptic pseudarthrosis were culture positive, the most common causative pathogens being *C. acnes* and CNS in 46.2% and 38.5% of the time, respectively. 26 In our institution, intraoperative cultures are performed in all revision cases for pseudarthrosis, even without preoperative suspicion of SSI. However, many surgeons still carry out intraoperative cultures only when infection is visually suspected preoperatively, which may lead to an underestimation of the true prevalence. 26

This study had several limitations. First, the major limitation was its single-center nature and the small number of patients, which limits the capacity to generate more generalized conclusions. Thus, a large population-based study is warranted. Then, it was a retrospective study, which may generate biases inherent to retrospective studies. Finally, because SSI was not suspected preoperatively, CRP was not available in our study. Consequently, we could not determine if ^18^ F-FDG PET/CT–CRP combined evaluation can potentially enhance diagnostic accuracy, as was the case for patients with three or more vertebral levels fixed in the study by Follenfant et al. 14

## Conclusion

In presumed aseptic pseudarthrosis after spinal fusion, ^18^ F-FDG PET/CT offers good specificity (75%) but low sensitivity (50%) to identify occult SSI, mostly caused by low-virulence bacteria *C. acnes* (75%). The unexpectedly high prevalence (56%) of SSI caused by low-virulence bacteria found in our presumed aseptic cohort of patients supports the utility of systematic intraoperative cultures in revision cases for pseudarthrosis.
